# Alectinib after failure to crizotinib in patients with ALK-positive non-small cell lung cancer: results from the Spanish early access program

**DOI:** 10.18632/oncotarget.28244

**Published:** 2022-06-15

**Authors:** Reyes Bernabé-Caro, Pilar Garrido, Rosario García-Campelo, Ramón Palmero, Ángel Artal, Cristina Bayona, Delvys Rodríguez-Abreu, Marta López-Brea, Alfredo Paredes, David Vicente, José Miguel Sánchez Torres, Margarita Majem, Pilar Diz, Rocío Gordo, Margarita Coca, Javier de Castro

**Affiliations:** ^1^Department of Medical Oncology, Hospital Virgen del Rocío, Sevilla, Spain; ^2^Department of Medical Oncology, Hospital Universitario Ramon y Cajal, Madrid, Spain; ^3^Department of Medical Oncology, Hospital Universitario A Coruña, A Coruña, Spain; ^4^Department of Medical Oncology, ICO Bellvitge, Hospitalet Llobregat, Barcelona, Spain; ^5^Department of Medical Oncology, Hospital Universitario Miguel Servet, Zaragoza, Spain; ^6^Department of Medical Oncology, Hospital General Yague, Burgos, Spain; ^7^Department of Medical Oncology, Hospital Insular de Gran Canaria, Las Palmas de Gran Canaria, Spain; ^8^Department of Medical Oncology, Hospital Marqués de Valdecilla, Santander, Spain; ^9^Department of Medical Oncology, Hospital Universitario Donostia, Donostia-San Sebastián, Spain; ^10^Department of Medical Oncology, Hospital Universitario Virgen Macarena, Sevilla, Spain; ^11^Department of Medical Oncology, Hospital Universitario La Princesa, Madrid, Spain; ^12^Department of Medical Oncology, Hospital de la Santa Creu i Sant Pau, Barcelona, Spain; ^13^Department of Medical Oncology, Complejo Asistencial Universitario de León, León, Spain; ^14^Roche Farma, S.A., Madrid, Spain; ^15^Department of Medical Oncology, Hospital Universitario La Paz, Madrid, Spain

**Keywords:** ALK-positive NSCLC, ALK inhibitor, crizotinib, alectinib, unselected patient

## Abstract

This retrospective observational study analyzed the clinical characteristics, treatment patterns and outcomes of 120 patients with advanced ALK-positive non-small-cell lung cancer (ALK+ NSCLC) according to data collected between November 2019 and October 2020 in 38 Spanish hospitals. Patients had progressed after 1–5 prior treatment lines (which included crizotinib in any prior line) and received subsequent therapy with alectinib in a local expanded access program. Median age was 58.7 years, 50% of patients were female, 64.1% had ECOG PS of 0–1, 85% presented stage IV, 95% had adenocarcinoma histology and 20.8% had brain metastases. After a median 9.6 months of alectinib treatment, objective response rate (ORR) was 54.5%, disease control rate (DCR) was 80%, median progression-free survival (PFS) was 9.4 months and median overall survival (OS) was 24.1 months. Patients with brain metastases achieved an intracranial DCR of 71.4%. Adverse events (AEs) were reported in 35.8% of patients (14.2% of AEs were grade ≥3). Over 40% of patients received some treatment after alectinib, most frequently lorlatinib (65.2%) and brigatinib (32.6%). This study provides information on real-world treatment patterns and confirms the tolerability and prolonged PFS and OS observed with alectinib in clinical trials, in unselected pretreated patients with advanced ALK+ NSCLC.

## INTRODUCTION

Rearrangement in the anaplastic lymphoma kinase (ALK) gene occurs in 2–5% of non-small-cell lung cancer (NSCLC) cases and leads to constitutive activation of the ALK kinase, which promotes ALK-driven tumorigenesis [1]. ALK-positive NSCLC (ALK+ NSCLC) is characterized by frequent dissemination to the central nervous system (CNS) [2–4].

Following its approval in 2011, crizotinib became the standard first-line treatment for advanced ALK+ NSCLC. However, most patients treated with this drug relapsed within 1 year, due mainly to the development of crizotinib resistance or poor CNS penetration [5, 6]. The median progression-free survival (PFS) with first-line crizotinib was 10.9 months [5]. Second-generation ALK inhibitors (such as ceritinib, alectinib and brigatinib) emerged and were initially approved for use after crizotinib progression. Ceritinib was approved in 2015 for use in ALK+ NSCLC patients who had progressed on crizotinib. Alectinib is a highly specific ALK tyrosine kinase inhibitor (TKI) that has shown activity against a broad range of mutations responsible for crizotinib resistance, and has demonstrated both clinical systemic and CNS efficacy as well as a good safety profile in clinical trials. Pooled data from the 2 pivotal multicenter phase II studies of alectinib (NP28761 and NP28673) [7, 8] in ALK+ NSCLC patients who had progressed on, or were intolerant to, crizotinib, confirmed the efficacy of alectinib over long follow-up periods [9]. The authors reported an objective response rate (ORR) of 51.3%, disease control rate (DCR) of 78.8%, median progression-free survival (PFS) of 8.3 months and median overall survival (OS) of 29.1 months, with a good safety profile [9, 10].

The phase III ALUR study, designed to compare the efficacy and safety of alectinib versus standard chemotherapy in 107 patients previously treated with platinum-based doublet chemotherapy and crizotinib, showed significantly improved systemic efficacy with a median investigator-assessed PFS of 9.6 months with alectinib versus 1.4 months with chemotherapy (hazard ratio [HR]: 0.15, 95% confidence interval [CI]: 0.08–0.29, *P* < 0.001). Alectinib showed clinically relevant superiority to chemotherapy for intracranial disease compared to chemotherapy, along with a favorable safety profile. The CNS ORR was significantly higher with alectinib (54.2%) versus chemotherapy (0%; *P* < 0.001). Grade ≥ 3 adverse events (AEs) were more common with chemotherapy (41.2%) than with alectinib (27.1%), while the incidence of AEs leading to discontinuation was lower with alectinib [11]. Final data from the ALUR trial confirmed the primary analysis (PFS of 10.9 months with alectinib versus 1.4 months with chemotherapy [HR: 0.2, 95% CI: 0.12–0.33,; *P* < 0.001) [12].

In the phase III ALEX trial, which compared alectinib with crizotinib as first-line treatment in 303 adult patients with advanced ALK+ NSCLC, alectinib demonstrated superior PFS, CNS activity, delayed CNS progression and lower toxicity than crizotinib, irrespective of prior CNS disease or radiotherapy, or type of ALK variant [13, 14]. Updated results from this trial have confirmed the superior investigator-assessed PFS, with a median of 34.8 months in the alectinib arm versus 10.9 months for crizotinib (HR 0.43, 95% CI; 0.32–0.58) and a 5-year OS rate of 62.5% with alectinib and 45.5% with crizotinib [15–17]. Alectinib superiority in independent review facility-assessed PFS versus crizotinib was confirmed in the Japanese phase III J-ALEX clinical trial, in which follow-up continues [18, 19]. Results from the phase III ALESIA study of first-line alectinib versus crizotinib in Asian patients with advanced ALK+ NSCLC confirmed the clinical benefit of alectinib in the first-line setting [20]. These findings changed the standard of care to alectinib as front-line therapy for patients with advanced ALK+ NSCLC [21].

Significant results obtained from clinical trials led to the first approval of alectinib for use in patients with advanced ALK+ NSCLC who relapsed or were intolerant to crizotinib, and in October 2017, the European Medicines Agency (EMA) recommended extending the indication for alectinib to treatment-naïve advanced ALK+ NSCLC patients [22].

Next-generation TKI are listed as the recommended first-line therapy for advanced ALK+ NSCLC [23, 24]. Indeed, the latest National Comprehensive Cancer Network (NCCN) guidelines recommend the use of alectinib, brigatinib, and lorlatinib as preferred first-line treatment options [25]. Overall, next-generation ALK TKIs (alectinib, ceritinib, brigatinib) have replaced the first-generation TKI crizotinib as first-line treatments for ALK+ NSCLC patients [17].

Despite the satisfactory outcomes demonstrated with alectinib in clinical trials and its extensive use in the first-line setting, there is a lack of real-world data on unselected populations, which could help to fill gaps in the literature.

In Spain, alectinib has been used after failure to crizotinib in advanced ALK+ NSCLC within an expanded access program (EAP). The present REALK study aimed to define this patient population and to assess the treatment patterns used in clinical practice and the associated clinical outcomes.

## RESULTS

### Patient clinical and pathophysiological characterization at diagnosis

All clinical and demographic features were assessed at different time points during the follow-up: at diagnosis of advanced ALK+ NSCLC; during treatments prior to alectinib treatment; at initiation, during and after alectinib treatment; and after subsequent lines of treatment. Just over half the patients (55.8%) had died at the time of data collection, mostly due to disease progression (91.0%). Table 1 summarizes the patient characteristics at diagnosis. Of 120 patients, 50% were female, mostly Caucasian (96.7%). Mean age at diagnosis of advanced disease was 58.7 years and 66.7% of patients were younger than 65 years. Most patients were non-smokers (45.8%), while 29.2% were former smokers and 25% were current smokers. Eighty-five percent of patients had stage IV disease and 20.8% presented brain metastases. Adenocarcinoma histology was reported in 95% of patients. ECOG PS 0 or 1 was reported in 25.8% and 38.3% of patients, respectively. Diagnosis of ALK translocation was confirmed using fluorescent *in situ* hybridization (FISH) or immunohistochemistry in 53.3% and 43.3% of patients, respectively. The percentage of patients with abnormal liver function tests based on altered levels of alanine transaminase (ALT), aspartate transaminase (AST), gamma-glutamyl transferase (GGT) or bilirubin at diagnosis were 8.3%, 5%, 16.7% and 4.2%, respectively.

**Table 1:** Demographic and clinical characteristics of patients included in the study at diagnosis of advanced NSCLC

Characteristics	Total patients (120)
**Sex, *n* (%)**	
Female	60 (50)
Male	60 (50)
**Age (years)**	
Mean (SD)	58.7 (12.9)
Median (min, max)	57.5 (49-69)
**Age distribution at diagnosis, *n* (%)**	
< 65 years	80 (66.7)
≥ 65 years	40 (33.3)
**Ethnicity, *n* (%)**	
Caucasian	116 (96.7)
Non-Caucasian	4 (3.3)
**Smoking status at diagnosis, *n* (%)**	
Active smoker	30 (25)
Former smoker	35 (29.2)
Non-smoker	55 (45.8)
**Disease stage, *n* (%)**	
IIIB	18 (15)
IV	102 (85)
**Histology, *n* (%)**	
Adenocarcinoma	114 (95)
Large cell carcinoma	1 (0.8)
Squamous-cell carcinoma	3 (2.5)
Undifferentiated	1 (0.8)
Other	1 (0.8)
**ECOG PS at diagnosis, *n* (%)**	
ECOG 0	31 (25.8)
ECOG 1	46 (38.3)
ECOG 2	10 (8.3)
ECOG 3	0 (0)
ECOG 4	1 (0.8)
Unknown	32 (26.7)
**Metastasis at advanced diagnosis, *n* (%)**	
Yes	102 (85)
No	18 (15)
**Brain metastasis at diagnosis, *n* (%)**	
Yes	25 (20.8)
No	95 (79.2)

^*^1 patient with unknown date of ALK-translocation diagnosis. Abbreviations: ECOG PS: ECOG performance status; min: minimum; max: maximum; SD: standard deviation.

### Patient characteristics and prior treatment regimens at initiation of alectinib

At initiation of alectinib therapy, 38.3% and 15% of patients had known ECOG PS 0 and 1, respectively (Table 2). As per protocol, all patients included in the study had been treated with at least one prior line of crizotinib. Almost half the patients (46.7%) had been treated with only 1 prior treatment line, 38.3% had received 2 previous treatment lines and 15.1% received ≥3 lines.

**Table 2:** Patient characteristics at initiation and during follow up of alectinib treatment

Characteristics	Total patients	
**Lines of treatment prior to alectinib, *n* (%)**	**120 (100)**	
1 line	56 (46.7)	
2 lines	46 (38.3)	
3 lines	8 (6.7)	
4 lines	8 (6.7)	
5 lines	2 (1.7)	
**Reason for alectinib initiation^a^, *n* (%)**	**120 (100)**	
Toxicity of previous treatments	14 (11.7)	
Disease progression	101 (84.2)	
Other	5 (4.2)	
**Total daily dose of alectinib (mg)**		
Median (min, max)	1,200 (600, 1,200)	
**Duration of alectinib treatment^b^ (months)**		
Mean (SD; 95% CI)	13.9 (12.3; 11.7–16)	
Median (IQR; min, max)	9.6 (3.1–25.2; 0.2, 39.2)	
**Reason for alectinib discontinuation, *n* (%)**	**84 (100)**	
Toxicity	2 (2.4)	
Disease progression	62 (73.8)	
Death/Other^c^	15 (17.9)/5 (6)	
**Best ECOG PS, *n* (%)**	**At initiation**	**During treatment**
ECOG 0	18 (15)	40 (33.3)
ECOG 1	46 (38.3)	50 (41.7)
ECOG 2	16 (13.3)	10 (8.3)
ECOG 3	2 (1.7)	4 (3.3)
ECOG 4	1 (0.8)	0 (0)
Unknown	37 (30.8)	16 (13.3)

^a^Impaired renal function (1 patient); CNS progression and medical decision (1 patient); Due to a mycetoma, 1 patient was treated with voriconazole, which interacts negatively with crizotinib; Investigator’s criteria (1 patient); Pneumonitis (1 patient); ^b^Difference in months between Start date and End date (for ongoing treatments, date of inclusion/date of exitus/date of last contact has been considered as End date); ^c^Clinical disease (1 patient); Resistance mutation (1 patient); Respiratory failure (1 patient); Clinical deterioration (1 patient); Maximum benefit (1 patient). Abbreviations: CI: confidence interval; CNS: central nervous system; ECOG PS: Eastern Cooperative Oncology Group performance status; IQR: interquartile range (25–75); min: minimum; max: maximum; SD: standard deviation.

In the first and second lines of treatment, crizotinib was the preferred therapy (59.2% and 34.2% of patients, respectively) followed by chemotherapy (42.5% and 8.3% of patients, respectively), while the use of ceritinib was very limited (5.8% and 4.2%, respectively). After crizotinib, chemotherapy was the most widely used therapy (45.8% of patients) in all previous treatment lines, followed by ceritinib (13.3%) and brigatinib (6.7%) (Supplementary Table 1). The mean duration of treatment (DOT) prior to alectinib was 9.1 months (95% CI: 7.8–10.3), with a median of 7.5 months (IQR: 2.8–14.4). Most patients (86.7%) discontinued prior treatments due to disease progression. Indeed, this was the main cause of crizotinib discontinuation (82.5% of patients), although 13.3% of patients discontinued due to toxicity (Supplementary Table 1). At initiation of alectinib treatment, 12.5%, 8.3% and 18.3% of patients presented altered ALT, AST or GGT values, respectively; in addition, 3.3% presented abnormal bilirubin values (data not shown).

### Patient characteristics during follow-up of alectinib treatment

Most patients (84.2%) initiated alectinib treatment due to disease progression after failure to previous treatment lines (Table 2). Mean DOT with alectinib was 13.9 months (95% CI: 11.7–16) with median DOT of 9.6 months (IQR: 3.1–25.2) and maximum DOT of 39.2 months. Patients received a median daily dose of alectinib of 1200 mg/day (600 mg twice daily as recommended (IQR: 1200–1200) with a minimum dose of 600 mg/day (300 mg twice daily). During follow-up of patients under alectinib treatment, 33.3% presented an ECOG PS 0, 41.7% had ECOG PS 1 and 8.3% had ECOG PS 2 (Table 2). According to the liver function tests, 20.8% to 31.7% of the patients presented altered levels of ALT or AST and almost 21% of patients had altered GGT or bilirubin values. The main reason for alectinib discontinuation was disease progression (73.8% of patients).

### Patient characteristics after alectinib and subsequent treatment regimens

Of 120 patients, 46 (38.7%) followed subsequent treatments after alectinib. In this subset of patients, lorlatinib was the most frequently prescribed therapy (65.2% patients), followed by brigatinib (32.6%) and chemotherapy (28.3%) (Table 3). One single patient may have simultaneously received more than 1 of these treatments. Mean duration of subsequent treatments after alectinib was 5.3 months (95% CI: 3.8–6.7) and median DOT was 3.5 months (IQR: 1.7–5), with a maximum duration of 18.1 months. The most common reason for treatment discontinuation was disease progression (56.5% of patients). ECOG PS was 0 for 26.1% of patients, 1 for 39.1%, 2 for 15.2%, and 3 for 6.5% (Table 3).

**Table 3:** Characteristics of patients who followed subsequent treatments after alectinib regimen

Characteristics	Total patients	Patients under subsequent treatments
**Type of treatment, *n* (%)**	120 (100)	48 (100)
Chemotherapy	13 (10.8)	13 (28.3)
Immunotherapy^a^	5 (4.2)	5 (10.9)
Brigatinib	15 (12.5)	15 (32.6)
Lorlatinib	30 (25)	30 (65.2)
Other^b^	2 (1.7)	2 (4.3)
**Best ECOG PS, *n* (%)**	120 (100)	48 (100)
ECOG 0	12 (10)	12 (26.1)
ECOG 1	18 (15)	18 (39.1)
ECOG 2	7 (5.8)	7 (15.2)
ECOG 3	7 (5.8)	3 (6.5)
Unknown	17 (14.2)	17 (37)
**Reason for discontinuation, *n* (%)**		
Toxicity	3 (2.5)	3 (6.5)
Disease progression	26 (21.7)	26 (56.5)
Death	2 (1.7)	2 (4.3)
Other^b^	12 (10)	12 (26.1)
**Duration of treatment, (months)^c^**		
Mean (SD; 95% CI)	5.3 (4.8; 3.8–6.7)	
Median (IQR; min, max)	3.5 (1.7-7.5; 0.2, 18.1)	
**TTP (months)**		
Mean (SD; 95% CI)	4.4 (3.3; 3–5.7)	
Median (IQR; min, max)	3.7 (1.9–6; 0.9–13.8)	

^a^Nivolumab (2 patients); Pembrolizumab (2 patients); Atezolizumab (1 patient); ^b^Alectinib (1 patient); crizotinib (1 patient); ^c^ For ongoing treatments, the date of inclusion/date of exitus/date of last contact has been considered as End date. Abbreviations: CI: confidence interval; ECOG PS: ECOG performance status; IQR: interquartile range (25–75); min: minimum; max: maximum; SD: standard deviation; TTP: time to progression.

### Characterization of metastases and patterns of disease progression

At diagnosis of advanced ALK+ NSCLC, the most common sites of metastases were the lung (50.0%) and bone (46.1%), followed by the CNS (24.5%) and liver (16.7%) (Table 4).

**Table 4:** Characteristics of the extension of the disease in patients with advanced NSCLC treated previously with crizotinib at diagnosis, during crizotinib, and during alectinib and subsequent treatments

Patients, *n*	Diagnosis	Crizotinib in previous lines			Prior to alectinib	During alectinib	Subsequent therapies
	102	Total 99	1st line 54	2nd line 36	104	62	26
**TTP (months)**							
Mean (SD) (95% CI)	– –	12.5 (10.1) (10.5–14.5)	10.6 (7.3) (8.6–12.7)	13.2 (10.9) (9.5–16.8)	10.7 (7.9) (9.13–12.2)	7.8 (6) (6.3–9.3)	4.4 (3.3) (3–5.7)
Median (IQR) (min, max)	– –	9.4 (4.8–17.8) (0.4–51)	9 (4.3–16.9) (0.4–26.8)	8.9 (5.5–20.2) (0.7–44)	9 (3.9–16.8) (0.4, 44)	6.4 (2.6–10.7) (0.3, 27.5)	3.7 (1.9–6) (0.9, 13.8)
**Type, *n* (%)**		**99 (100)**	**54 (100)**	**36 (100)**	**104 (100)**	**62 (100)**	**26 (100)**
Local recurrence	– –	27 (27.3)	15 (27.8)	9 (25)	34 (32.7)	12 (19.4)	9 (34.6)
Regional recurrence	– –	13 (13.1)	10 (18.5)	2 (5.6)	13 (12.5)	7 (11.3)	2 (7.7)
Distant recurrence	– –	58 (58.6)	29 (53.7)	24 (66.7)	69 (66.3)	43 (69.4)	16 (61.5)
**Location, *n* (%)**							
CNS metastases	25 (24.5)	41 (41.4)	24 (44.4)	13 (36.1)	47 (45.2)	24 (38.7)	4 (15.4)
Liver	17 (16.7)	16 (16.2)	7 (13)	8 (22.2)	23 (22.1)	13 (21)	5 (19.2)
Bone	47 (46.1)	20 (20.2)	11 (20.4)	9 (25)	29 (27.9)	18 (29)	12 (46.2)
Lung	51 (50)	38 (38.4)	23 (42.6)	11 (30.6)	46 (44.2)	30 (48.4)	15 (57.7)
Adrenal gland	0 (0)	3 (3)	3 (5.6)	0 (0)	5 (4.8)	0 (0)	1 (3.8)
Other	38 (37.3)	15 (15.2)	10 (18.5)	5 (13.9)	27 (26)	13 (21)	8 (30.8)

A single patient may be classified simultaneously in more than 1 category and report more than 1 tumour location. Not all patients included in the study had data on extension of disease available, thus N may differ from other tables. Abbreviations: CI: confidence interval; CNS: central nervous system; IQR: interquartile range (25–75); min: minimum; max: maximum; SD: standard deviation; TTP: time to progression.

During treatments prior to alectinib, metastases were mostly located in the CNS and lung (45.2% and 44.2% of patients, respectively) followed by bone (27.9%) and liver (22.1%) (Table 4). Mean time to progression with prior treatment lines was 10.7 months (95% CI: 9.13–12.2) and median time to progression was 9.0 months (IQR: 3.9–16.8) (Supplementary Table 1). CNS metastases were identified in 41.4% of patients who had received crizotinib prior to alectinib therapy (Table 4). According to the line of treatment in which crizotinib was used, 44.4% and 36.1% of patients treated with crizotinib in the first and second line, respectively, reported CNS metastases.

During alectinib treatment, 69.4% of patients had distant recurrences (Table 4). Lung metastases were reported in 48.4% of patients, followed by CNS metastases in 38.7% of patients; bone and liver metastases were reported in 29.0% and 21.0% of patients, respectively. Progression was confirmed in 79% of patients during the first year and 21% of patients progressed during the second year. Mean time to progression was 7.8 months (95% CI: 6.3–9.3) with a median of 6.4 months (IQR: 2.6–10.7) (Table 4).

### Characterization and management of CNS metastases

CNS metastases were confirmed in a high number of advanced ALK+ NSCLC patients, irrespective of the time of analysis (Supplementary Table 2). They were confirmed in a total of 25 patients at diagnosis, using magnetic resonance imaging (MRI; 68.0%) or computed axial tomography (CT) (36.0%).

Most CNS metastases (≥50%) were reported as symptomatic at all analysed time points and as multiple lesions. Leptomeningeal carcinomatosis was very rare at diagnosis, and before initiation of and during alectinib treatment. However, the presence/absence of leptomeningeal carcinomatosis was not characterized in a high percentage of patients (around 40%), and it was not characterized during subsequent therapies (Supplementary Table 2).

Approximately 50% of patients with CNS metastases received some type of local treatment for the management of brain metastasis at diagnosis, during alectinib treatment and during subsequent therapies (Supplementary Table 2). Only 29.8% of patients were treated locally for CNS metastases before alectinib initiation. The most common local treatments at diagnosis were whole-brain radiotherapy (WBRT) (50%) or radiosurgery (50%). During alectinib treatment and subsequent lines of therapy, radiotherapy was the most common therapeutic choice (>60%). The use of corticosteroids was reported in 23.2% of patients with CNS metastases at diagnosis, and increased during alectinib treatment (10 out of 12 patients: 83.3%) (Supplementary Table 2).

During alectinib treatment, the percentage of patients with CNS metastases decreased from 45.2% to 38.7% (Table 4). CNS ORR during alectinib treatment was not available for the total number of patients (data collected from a sample of 21 patients are summarized in Supplementary Table 3). In evaluable patients, the ORR was 28.6% and DCR was 71.4%. The mean duration of response (DOR) was 11 months (95% CI: 2–7) and median DOR was 10.2 months (IQR: 4.4–13.4). Based on 10 patients with measurable metastases, ORR was 30.0% and DCR was 70.0%. Mean time to first response was 4.1 months (95% CI: 1.9–6.2) and median time to first response was 2.6 months (IQR: 1.3–3.5), with a maximum of 17.1 months. During alectinib treatment, mean time to first response or to reduction of corticosteroids was 4.1 ± 4.6 months (95%CI: 1.9–6.2), with a median of 2.6 months (IQR: 1.3–3.5) (Supplementary Table 2).

### Secondary outcomes after alectinib use in a real-world clinical practice setting

The effectiveness of alectinib in real-world clinical practice was assessed as a secondary endpoint in this study. Response rates, PFS and OS were assessed.

The mean time from administration of the first dose of alectinib to the first response was 2.6 months (95% CI: 2.1–3) (Table 5). Best responses were reported in 110 patients, with an ORR of 54.5% and DCR of 80%. The mean time from the first dose of alectinib to the best response achieved was 4.8 ± 5.1 months (95% CI: 3.8–5.8). Mean DOR was 7.5 months (95% CI: 5.6–9. 4) (Table 5).

**Table 5:** First and best responses achieved during alectinib treatment

**First response, *n* (%)**	**Patients 111 (100)**
Complete response (CR)	2 (1.8)
Partial response (PR)	55 (49.5)
Stable disease (SD)	29 (26.1)
Progressive disease (PD)	15 (13.5)
Not evaluable (NE)	10 (9)
**Time to first response (months)**	**Valid *N* (107)**
Mean (SD; 95% CI)	2.6 (2.1; 2.1–3)
Median (IQR; min, max)	2.4 (1.5–3; 0, 14.6)
**Best response, *n* (%)**	**110 (100)**
Complete response (CR)	5 (4.5)
Partial response (PR)	55 (50)
Stable disease (SD)	28 (25.5)
Progressive disease (PD)	14 (12.7)
Not evaluable	8 (7.3)
**Objective response rate (ORR), *n* (%)**	**110 (100)**
CR + PR	60 (54.5)
**Disease control rate (DCR), *n* (%)**	**110 (100)**
CR + PR + SD	88 (80)
**Time to best response (months)**	**Valid *N* (106)**
Mean (SD; 95% CI)	4.8 (5.1; 3.8–5.8)
Median (IQR; Min, max))	2.8 (1.9–5.9; 0.1, 21.2)
**Duration of response (DOR, months)**	**Valid *N* (37)**
Mean (SD; 95% CI)	7.5 (5.8; 5.6–9.4)
Median (IQR; min, max)	5.3 (3.4–9.2; 0.2, 22.4)

Abbreviations: CI: confidence interval; IQR: interquartile range (25–75); min: minimum; max: maximum; SD: standard deviation.

**Figure 1:**
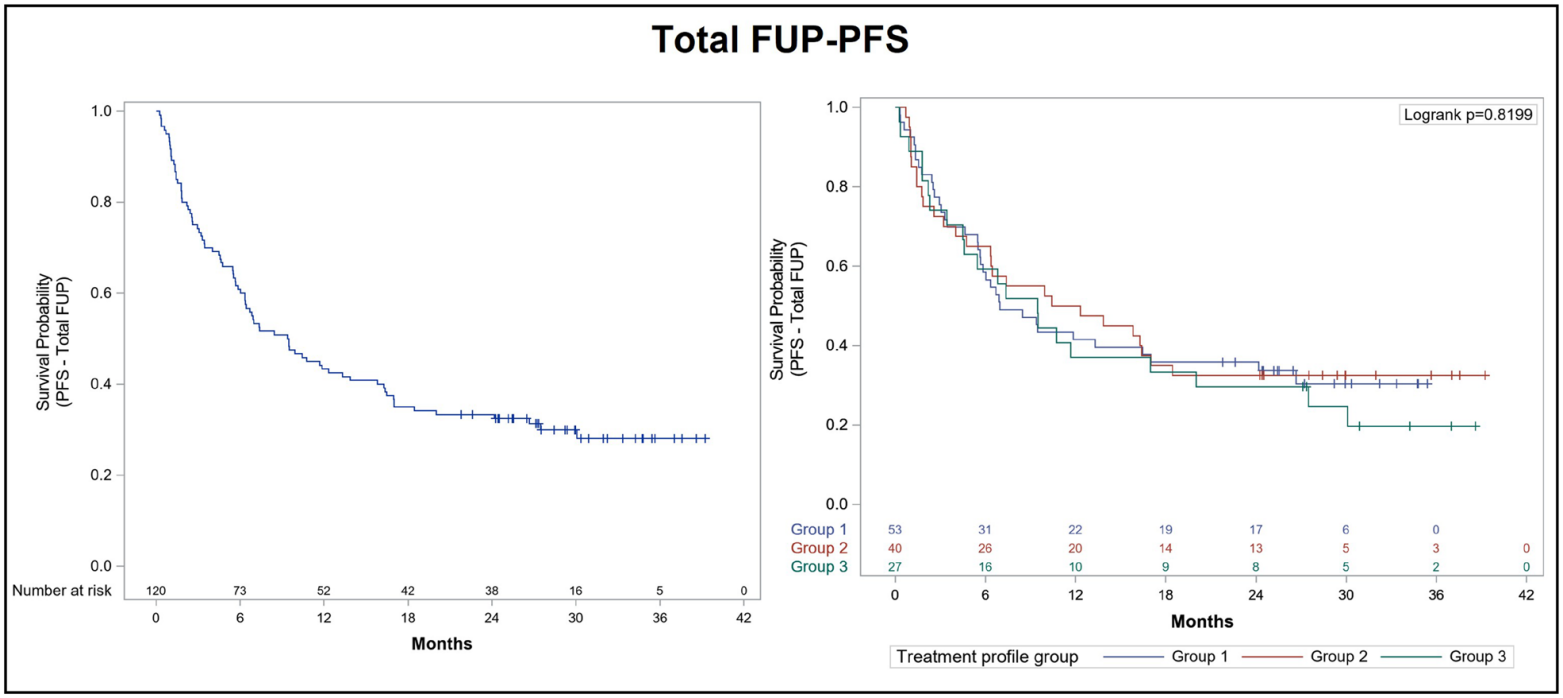
Kaplan-Meier curve for progression-free survival after total follow-up of ALK+ NSCLC patients treated with alectinib. Progression free survival (PFS) was analyzed after total follow-up (FUP) according to prior lines of treatment in the overall population (left panel) and in each subgroup of the effectiveness population (right panel): Group 1 (53 patients previously treated with crizotinib only), group 2 (40 patients who had received previous lines of crizotinib and chemotherapy), and group 3 (27 patients who had received prior lines of crizotinib and other ALK inhibitors, with or without chemotherapy).

**Figure 2:**
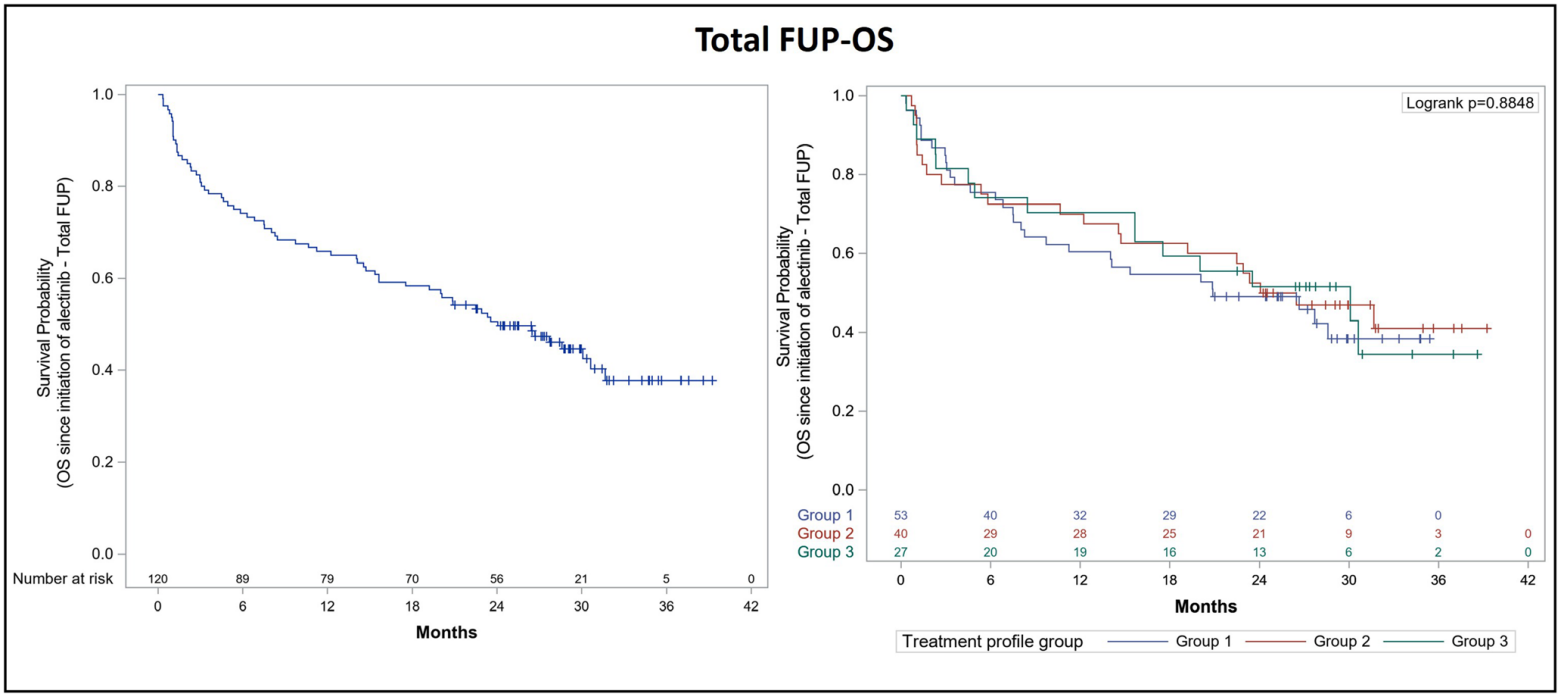
Kaplan-Meier curve for overall survival after total follow-up of ALK+ NSCLC patients treated with alectinib. Overall survival (OS) was analyzed after total follow-up (FUP) according to prior lines of treatment in the overall population (left panel) and in each subgroup of the effectiveness population (right panel): Group 1 (53 patients previously treated with crizotinib only), group 2 (40 patients who had received previous lines of crizotinib and chemotherapy), and group 3 (27 patients who had received prior lines of crizotinib and other ALK inhibitors, with or without chemotherapy).

Following the initiation of alectinib therapy, 70% of patients progressed or died during the overall follow-up period. Mean duration of total follow-up for the PFS analysis was 29.9 months (95% CI: 28.3–31.6) and 1-year and 2-year PFS rates were 43.3% and 33.3%, respectively. Median PFS was 9.4 months (95% CI: 6.3–13.8). Mean duration of total follow-up for the OS analysis was 29.2 months (median OS follow-up was 24.1 months [95% CI: 15.6–31.7]). Median OS after total follow-up was 24.1 months (95% CI: 15.6–31.7). The 1-year and 2-year OS rates were 65.8% and 50.8%, respectively. Since the diagnosis of advanced NSCLC, 42.4% of patients were still alive, mean follow-up duration for OS was 59.6 months (95% CI: 53.2–66.1) and median OS was 43.3 months (95% CI: 33.4–59.9). The Kaplan-Meier curves for PFS and OS after total follow-up are presented in Figures 1 and 2, respectively.

We also performed an exploratory analysis in order to obtain a clearer picture of alectinib effectiveness according to previous lines of treatment. Thus, we classified the effectiveness population into three groups of patients: Group 1 (53 patients previously treated with crizotinib only), group 2 (40 patients who had received previous lines of crizotinib and chemotherapy), and group 3 (27 patients who had received prior lines of crizotinib and other ALK inhibitors, with or without chemotherapy). The analysis of PFS according to previous lines of treatment confirmed that patients included in group 1 reached a median PFS of 7 months (95% CI: 5.5–17), while median PFS was 11.4 months (95% CI: 4.7–17) for group 2 and 9.5 months (95% CI: 3.4–20) for group 3 (Table 6). The 1-year and 2-year PFS rates were 41.5% and 35.8%, respectively, for patients in group 1, 50.0% and 32.5%, respectively, for group 2 patients, and 37% and 29.6%, respectively, for patients in group 3 (Figure 1). Following the diagnosis of advanced NSCLC, 1-year and 2-year OS rates were 77.4% and 62.3% for group 1, 90% and 80% for group 2, and 96.3% and 81.5% for group 3 patients, respectively (Figure 2).

**Table 6:** Type, severity and management of AEs reported during alectinib treatment

	Total	Grade <3	Grade ≥3
**Patients with AEs, *n* (%)**	43 (35.8)	18 (15)	17 (14.2)
**Reported AEs, *n* (%)**	**70 (100)**	**27 (38.6)**	**22 (31.4)**
Serious AEs	23 (32.9)	4 (14.8)	10 (45.5)
AEs related to alectinib	33 (47.1)	17 (63)	7 (31.8)
Not evaluable	8 (11.4)	3 (11.1)	1 (4.5)
**Action taken, *n* (%)**			
No action taken	19 (27.1)	3 (11.1)	7 (31.8)
Study drug delayed	2 (2.9)	0 (0.0)	2 (9.1)
Study drug withheld temporarily	18 (25.7)	13 (48.1)	3 (13.6)
Dose reduction	30 (42.9)	16 (59.3)	5 (22.7)
Study drug interrupted permanently	4 (5.7)	0 (0.0)	4 (18.2)

Abbreviations: CI: confidence interval; IQR: interquartile range (25–75); SD: standard deviation.

Total follow-up of patients since the initiation of alectinib found OS rates of 43.4%, 45% and 44.4% for groups 1, 2 and 3, respectively.

A total of 43 patients (35.8%) reported 70 AEs during the treatment with alectinib. Grade ≥3 AEs were reported by 14.2% of patients (Table 6). A total of 33 AEs (47.1%) were related to alectinib treatment (63.0% of grade <3 AEs and 31.8% of grade ≥3 AEs). Dose reduction was applied for 42.9% of AEs while only 5.7% of total AEs led to permanent discontinuation. Most patients (75.5%) achieved total recovery.

Classification and proportion of AEs according to MedDRA are provided in Supplementary Table 4. The most frequent AEs were increased transaminase levels (5.8%, including ALT, AST, GTT and others), dyspnea (4.2%), asthenia (2.5%), fatigue (2.5%), increased serum bilirubin levels (2.5%), myalgia/myositis (2.5%) and vomiting (1.7%).

## DISCUSSION

The availability of next-generation ALK inhibitors has widened the therapeutic landscape for ALK+ NSCLC patients. However, further information on real-world treatment patterns is still needed in this context, as some patient subpopulations are underrepresented in clinical studies. This study gathered information from patients with advanced ALK+ NSCLC treated in 38 healthcare centers, who were included in an EAP of alectinib after progression on crizotinib, among other prior therapies. Current clinical guidelines recommend the use of next-generation TKIs (in particular, alectinib) as first-line therapy for advanced ALK+ NSCLC. Our aim was to provide a complete clinical characterization of an ALK+ NSCLC population of patients included in these types of access programs, and to assess the outcomes of advanced ALK+ NSCLC patients under sequential lines of treatment. Although available information was not complete in some cases, we observed some interesting trends in real-world clinical practice from these analyses.

The baseline characteristics of the study patient population were consistent with those reported in lung cancer patients with ALK-rearranged tumors, who are often younger than the general lung cancer population and present light or non-smoking habits and adenocarcinoma histology [26]. Our patient sample had similar features to other ALK+ NSCLC populations included in several real-world studies [27–32]: half the patients were female, with a mean age below 60 years, most were non-smokers, and they presented stage IV disease and adenocarcinoma histology. ECOG PS 0 and 1 were recorded in a high percentage of patients and 20.8% presented brain metastases at diagnosis. All patients included in this study had received crizotinib in a treatment line prior to alectinib (mostly in the first and second line). The most common type of non-targeted therapy received prior to alectinib was chemotherapy (45.8% of patients), while ceritinib and brigatinib were used to a lesser extent. Immunotherapy was used in the first, second and third line of treatment, but only in a very small proportion of patients (included in 1.7% of patients who may also have received other targeted therapies). This low frequency of prescription of immunotherapy could be explained by the poor and limited evidence of efficacy of immunotherapy in the treatment of ALK+ NSCLC patients [33–35]. The median duration of previous lines of treatment was 9.6 months, in the range of the DOT with some agents such as crizotinib, as reported in registrational trials [5].

With the approval of new second- and third-generation ALK inhibitors (alectinib, brigatinib and lorlatinib, among others), the therapeutic options for first and subsequent lines of treatment were expanded, offering clinicians a wider repertoire of therapeutic agents for initial treatment and the possibility of choosing between different treatment sequences for advanced ALK+ NSCLC [22, 36–38]. Current treatment guidelines recommend the use of multiple ALK inhibitors in the advanced setting [24, 39]. In clinical practice, next-generation ALK inhibitors (alectinib, ceritinib and brigatinib) have generally replaced the first-generation TKI crizotinib as first-line treatment. This has been possible due to the improved pharmacological properties of next-generation versus first-generation ALK TKIs, which include greater potency/selectivity, CNS penetration and targeting of resistant mutations [40, 41]. Third-generation inhibitors such as lorlatinib [41] were designed to overcome resistance to first- and even second-generation ALK-inhibitors [7, 8]. In our study, patients who followed subsequent lines of therapy after alectinib treatment were mostly prescribed lorlatinib (65.2% of 46 patients) followed by brigatinib (32.6%). Non-targeted therapies such as chemotherapy were prescribed to 28.3% of patients, while 10.9% of patients were treated with immunotherapy. These changes in treatment patterns reflect the emergence of new therapeutic alternatives and the update to recommendations in clinical guidelines that occurred between the initial NSCLC diagnosis and the termination of the study.

The use of sequential lines of active therapies resulted in an increase in the percentage of patients with ECOG PS 0 and 1 (from 26% to 38% at baseline, to 38% to 53%, respectively). Alectinib treatment, which was mostly initiated after disease progression, was still able to provide patients with optimal performance status at similar rates (33% and 42%, respectively), which translated into patients’ perception of being able to perform regular activities with no difficulties. Alectinib treatment achieved partial response in half of patients, with an ORR of 54.5% and DCR of 80%. Following the initiation of alectinib, the total median OS (24.1 months; 95% CI: 15.6–31.7) and median PFS (9.4 months; 95% CI: 6.3–13.8) were found to be in the same range as the outcomes reported in the alectinib phase II trials (NP28761 and NP28673) and in the phase III ALUR trial in patients who were intolerant or progressed on crizotinib [9, 12]. Our OS data improve on the previously reported results of a French EAP of crizotinib for ALK+ NSCLC patients, who achieved a median OS of 16.6 months after initiation of crizotinib. This superiority of alectinib versus crizotinib in EAP is consistent with the reported superiority of alectinib versus crizotinib when used as first-line treatment in naïve ALK+ NSCLC patients [17, 42].

In our real-world study, the median OS following the diagnosis of advanced ALK+ NSCLC was 43.3 months (95% CI: 33.4–59.9) considering that all patients had received crizotinib prior to alectinib and more than 40% of patients had received chemotherapy prior to ALK-directed therapy. The updated data from the ALEX trial on alectinib in the first-line setting confirmed a 5-year OS rate of 62.5% (95% CI 54.3–70.8) with alectinib versus 45.5% (95% CI 33.6–57.4) with crizotinib (OS was not reached with alectinib versus 57.4 months with crizotinib; stratified HR 0.67, 95% CI: 0.46–0.98). Although a higher 2-year OS rate of 72.5% was reported in the alectinib arm compared to our recorded 2-year OS of 50.8%, all these data support the efficacy of next-generation ALK inhibitors in front-line therapy [17].

In this paper, we have described the efficacy of alectinib in terms of PFS or OS between patients grouped according to the number and type of previous treatments since initiation of alectinib. In this regard, the small sample size and intra-group heterogeneity should be taken into consideration. Moreover, the treatment sequences described herein are not representative of the current ALK treatment landscape due the subsequent incorporation of new generation ALK inhibitors. Identification of the most suitable subsequent lines of treatment for ALK+ NSCLC patients is critical in the therapeutic decision-making process. However, the optimal sequence of ALK inhibitors is still under consideration.

We were also able to observe a benefit of alectinib treatment in terms of CNS activity after several prior lines of therapy. CNS metastases were detected after first-line crizotinib therapy in 44.4% of patients. This is consistent with the reported data on progression of preexisting intracranial lesions or the development of new ones while patients were under crizotinib treatment, as a common manifestation of acquired resistance [5, 43]. In our study, the overall percentage of patients with CNS metastases fell to 38.7% after the initiation of alectinib treatment, irrespective of prior treatment lines. Alectinib showed a DCR of 71.4% and a median DOR of 10.2 months, which is in line with the superior CNS activity and significantly delayed CNS progression reported in clinical trials [13, 14]. It is also worth noting that over 50% of patients received WBRT to treat CNS metastases at diagnosis and before alectinib treatment, which could potentially lead to chronic toxicity. The choice of alectinib as a front-line treatment in this setting could reduce the need for WBRT, and therefore the accumulated toxicity, whilst not compromising efficacy. It is interesting to note that among symptomatic patients with CNS metastases, only 5 out of 23 patients were treated with corticosteroids before initiating alectinib treatment. This may be based on the high expectations regarding alectinib efficacy in the management of ALK+ NSCLC-associated CNS metastases according to the available evidence [44].

In our study, no restrictions were established regarding comorbidities, concomitant medications, number or type of previous treatment lines or the different clinical approaches selected to treat ALK+ NSCLC patients at the time of recruitment. Our real-world data confirm that alectinib was well tolerated in this unselected patient population. In this context, crizotinib-led sequences and the use of chemotherapy were the most common prior treatments. In terms of subsequent treatments after the alectinib regimen, lorlatinib and brigatinib were the preferred therapies. Our findings seem to support the feasibility of the sequential use of ALK inhibitors and the additional and clinically meaningful benefits of incorporating next-generation ALK inhibitors into the therapeutic armamentarium. More interestingly, our study reflects the real dynamic and changing patterns of treatment in a Spanish population of ALK+ NSCLC patients during routine clinical practice, and suggests good adherence of clinicians to the most up-to-date recommendations included in clinical guidelines and emerging therapeutic choices.

Our study has some limitations. First, the limited sample size did not allow us to obtain statistical significance in the subgroup analyses. However, and despite the limited number of patients, the absence of very stringent inclusion criteria makes our results more representative of a real-world setting, and reflect the heterogeneity of the population with ALK+ NSCLC. The limitation inherent to the analysis of data extracted from clinical practice documentation must also be taken into account, as medical information recorded for reasons other than research can be incomplete, and some data were probably lost during patient follow-up. It is important to highlight that most patients were polymedicated and, therefore, some results should be interpreted with caution.

In conclusion, the results obtained in alectinib clinical trials in patients with ALK+ NSCLC can be observed in a less selective patient population treated during routine clinical practice, even in patients who had been previously treated with several lines of therapy. The median OS achieved since diagnosis of advanced disease in patients treated with next-generation ALK inhibitors (such as alectinib) after crizotinib failure highlights the importance of early accurate diagnosis and access to next-generation agents for targeted therapy in molecularly-defined patient populations. The results of our study provide details on real-world treatment patterns when new ALK inhibitors are incorporated into the drug repertoire for ALK+ NSCLC patients, and provide initial information on the use of sequential ALK therapies. Further studies are warranted to determine the optimal sequence of treatments for ALK-rearranged NSCLC in terms of prolonging survival.

## MATERIALS AND METHODS

### Patients

Patients from 38 healthcare institutions, diagnosed with advanced/metastatic ALK+ NSCLC, aged ≥18 years who were treated with alectinib through an expanded access program (EAP) between April 1, 2017 and March 31, 2018, were enrolled in this study. All patients had progressed on crizotinib, which was a mandatory criterion to be included in the study, as this was the first approved indication for the use of alectinib by the European Medicine Agency (EMA. A total of 128 patients were selected. Patients with any medical or psychological condition that, in the physician’s opinion, might compromise the ability of the patient to give informed consent, were excluded. The final number of assessable patients meeting selection criteria was 120.

### Study design

This was a multicenter, retrospective patient chart review of observational (non-interventional) nature which aimed to characterize the clinical practice patterns in the management of advanced ALK+ NSCLC patients included within the EAP, as well as to describe the main patient outcomes. Available data from Case Report Forms (CRFs) were collected between November 2019 and November 2020, when the database was locked. Only data registered before the initiation of the study (the first Ethics Committee approval was obtained on June 26, 2019) were extracted, thus reflecting the routine management and regimen patterns used to treat the disease and avoiding interference with physicians’ clinical practice. Patients had to sign informed consent. To comply with the retrospective nature of the study, the Ethics Committee authorized data collection for patients who had died or had been lost to follow-up at the time of study initiation. The study was conducted in the medical oncology departments at 38 Spanish sites, seeking a homogeneous geographical distribution. Its multicenter nature aimed to improve the representativeness of the study population in Spain.

### Study endpoints

The demographic and clinical profile of ALK+ NSCLC patients within the EAP who received alectinib on crizotinib failure were determined as primary objectives. The clinical and demographic variables were collected at diagnosis, at inclusion in the EAP and initiation of alectinib treatment, and during subsequent regimens. Effectiveness of alectinib and management of metastases were assessed as secondary endpoints. Best response rates were defined by the response evaluation criteria in solid tumors (RECIST). Progression free survival (PFS) and overall survival (OS) were assessed at 1 and 2 years since initiation of alectinib treatment as well as overall median PFS/OS since the initiation of alectinib treatment to disease progression or death. Data on the diagnostic techniques used for CNS detection and characterization and type of local treatments used for metastases management were collected. Safety and tolerability of treatments were recorded throughout the study, and adverse events (AEs) were graded according to the National Cancer Institute´s Common Terminology Criteria for Adverse Events.

### Statistical analyses

Categorical variables were expressed as numbers and percentages, and continuous variables as mean and standard deviation (SD), or as medians and interquartile ranges (IQR) for variables without normal distribution. Distributions of PFS and OS were estimated using the Kaplan-Meier method. The Log-rank test was used for comparing the survival distribution of 2 or more groups. Normal distribution of the quantitative variables was tested using the Kolmogorov-Smirnov statistical test. For the association analyses between quantitative variables with normal distribution, the two-sided *t*-test was used, while the Mann-Whitney *U* test was used for the remaining variables. Fisher’s test or the Chi-square test was used to determine the association between qualitative variables. A *p*-value < 0.05 was considered statistically significant unless otherwise specified. The statistical analysis was performed using SAS software package, version 9.4.

## SUPPLEMENTARY MATERIALS



## Abbreviations

AE: adverse events
ALK+ NSCLC: advanced anaplastic lymphoma kinase (ALK)-positive non-small-cell lung cancer
ALT: alanine transaminase
AST: aspartate transaminase
CNS: central nervous system
CT: computed axial tomography
HR: hazard ratio
DCR: disease control rate
DOR: duration of response
DOT: duration of treatment
ECOG PS: Eastern Cooperative Oncology Group performance status
GGT: gamma-glutamyl transferase
IQR: interquartile range
MRI: magnetic resonance imaging
ORR: objective response rate
OS: overall survival
PFS: progression-free survival
TKI: tyrosine kinase inhibitor
