# Supplementary material for: Alectinib after failure to crizotinib in patients with ALK-positive non-small cell lung cancer: results from the Spanish early access program
Source: Oncotarget. 2022 Jun 15;13:812–27. doi: 10.18632/oncotarget.28244 (PMC9200434; doi:10.18632/oncotarget.28244)
Supplement: Supplementary file 1 [file oncotarget-13-28244-s001.pdf]

Alectinib after failure to crizotinib in patients with ALK-positive non-small cell lung cancer: results from the Spanish early access program

SUPPLEMENTARY MATERIALS

Supplementary Table 1: Type of treatment lines prior to alectinib, clinical features and proportion of patients

	TOTAL	1st line	2nd line	3rd line	4th line	5th line
**Type of treatment, *n* (%)**	**120 (100)**	**120 (100)**	**120 (100)**	**120 (100)**	**120 (100)**	**120 (100)**
Chemotherapy	55 (45.8)	51 (42.5)	10 (8.3)	5 (4.2)	3 (2.5)	1 (0.8)
Crizotinib	120 (100)	71 (59.2)	41 (34.2)	3 (2.5)	4 (3.3)	2 (1.7)
Ceritinib	16 (13.3)	2 (1.7)	7 (5.8)	5 (4.2)	2 (1.7)	0 (0)
Brigatinib	8 (6.7)	0 (0)	5 (4.2)	2 (1.7)	1 (0.8)	0 (0)
Lorlatinib	1 (0.8)	0 (0)	0 (0)	1 (0.8)	0 (0)	0 (0)
Other [a]	6 (5)	2 (1.7)	2 (1.7)	2 (1.7)	0 (0)	0 (0)
**Duration of treatment (months)[b]**						
Mean (SD; 95% CI)	9.1 (6.8; 7.8–10.3)	7.9 (8.1; 6.4–9.3)	10.9 (11.1; 8.1–13.6)	85 (6.3; 4.9–11.2)	16.5 (20.1; 2.1–31)	26.3 (12.9; -89.8–142.4)
Median (IQR; min, max)	7.5 (2.8–14.4; 0.4, 26.2)	4.8 (2–11.7; 03, 52)	7.2 (2.3–14.6; 0.5, 44)	6.2 (3.5–10.4; 0.7, 22.3)	9.5 (3.4–14.8; 03, 56)	26.3 (17.2–35.4; 17.1, 35.4)
**Reason for discontinuation, *n* (%)**	**120 (100)**	**120 (100)**	**120 (100)**	**120 (100)**	**120 (100)**	**120 (100)**
Disease progression	104 (86.7)	82 (68.3)	49 (40.8)	13 (10.8)	10 (8.3)	2 (1.7)
Toxicity	21 (17.5)	15 (12.5)	8 (6.7)	0 (0)	0 (0)	0 (0)
Other	36 (30)	29 (24.2)	8 (6.7)	5 (4.2)	0 (0)	1 (0.8)
**Reason for discontinuing chemotherapy, *n* (%)**	**55 (100)**	**51 (100)**	**10 (100)**	**--**	**--**	**--**
Disease progression	34 (61.8)	28 (54.9)	6 (60)	--	--	--
Toxicity	4 (7.3)	3 (5.9)	1 (10)	--	--	--
Other	26 (47.3)	23 (45.1)	4 (40)	--	--	--
**Reason for discontinuing crizotinib, *n* (%)**	**120 (100)**	**71 (100)**	**41 (100)**	**--**	**--**	**--**
Disease progression	99 (82.5)	54 (76.1)	36 (87.8)	--	--	--
Toxicity	16 (13.3)	12 (16.9)	4 (9.8)	--	--	--
Other	6 (5)	5 (7)	1 (2.4)	--	--	--
**Best ECOG PS**	**120 (100)**	**120 (100)**	**120 (100)**	**120 (100)**	**120 (100)**	**120 (100)**
ECOG 0	46 (38.3)	36 (30)	17 (14.2)	6 (5)	3 (2.5)	1 (0.8)
ECOG 1	64 (53.3)	51 (42.5)	31 (25.8)	8 (6.7)	6 (5)	1 (0.8)
ECOG 2	11 (9.2)	9 (7.5)	2 (1.7)	1 (0.8)	0 (0)	0 (0)
ECOG 3	1 (0.8)	0 (0)	1 (0.8)	0 (0)	0 (0)	0 (0)
ECOG 4	1 (0.8)	0 (0)	1 (0.8)	0 (0)	0 (0)	0 (0)
Unknown	32 (26.7)	28 (23.3)	12 (10)	3 (2.5)	1 (0.8)	0 (0)
**TTP (months)**						
Mean (SD; 95% CI)	10.7 (7.9; 9.1–12.2)	9.9 (8.5; 28–11.8)	12.1 (10.3; 9.2–15)	8.3 (6.4; 4.4–12.2)	15.31(17.6; 2.7–27.9)	34.5 (1.6; 19.7–49.4)
Median (IQR; min, max)	9 (3.9–16.8; 0.4, 44)	8.4 (3.4–14.8; 0.4, 52)	8.3 (5.1–17.8; 0.7, 44)	6.1 (4.9–10.2; 1.4, 21.4)	9.4 (3.9–14.8; 0.5, 51)	34.5 (33.4–35.7: 33.4, 35.7)
**Type of recurrence, *n* (%)**	**104 (100)**	**82 (100)**	**49 (100)**	**13 (100)**	**10 (100)**	**2 (100)**
Local	34 (32.7)	24 (29.3)	13 (26.5)	4 (30.8)	2 (20)	2 (100)
Regional	13 (12.5)	11 (13.4)	3 (6.1)	1 (7.7)	1 (10)	0 (0)
Distant	69 (66.3)	48 (58.5)	32 (65.3)	8 (61.5)	7 (70)	0 (0)

Location, *n* (%)	104 (100)	82 (100)	49 (100)	13 (100)	10 (100)	2 (100)
CNS metastases	47 (45.2)	28 (34.1)	16 (32.7)	5 (38.5)	6 (60)	0 (0)
Liver	23 (22.1)	14 (17.1)	10 (20.4)	2 (15.4)	0 (0)	0 (0)
Bone	29 (27.9)	19 (23.2)	16 (32.7)	3 (23.1)	1 (10)	0 (0)
Lung	46 (44.2)	36 (43.9)	16 (32.7)	7 (53.8)	3 (30)	0 (0)
Adrenal gland	5 (4.8)	4 (4.9)	1 (2)	0 (0)	0 (0)	2 (100)
Other	27 (26)	19 (23.2)	6 (12.2)	3 (23.1)	0 (0)	0 (0)

[a]Erlotinib (1 patient); gefitinib (1 patient); nintedanib (1 patient); pembrolizumab (3 patients); [b]Difference in months between Start date and End date (for ongoing treatments, the date of inclusion /date of exitus /date of last contact was considered as End date). Abbreviations: CI: confidence interval; CNS: central nervous system; ECOG PS: Eastern Cooperative Oncology Group performance status; IQR: interquartile range; SD: standard deviation; TTP: time to progression.

Supplementary Table 2: Types of treatment for CNS metastases according to the time of management

	At diagnosis	Before alectinib treatment	During alectinib treatment	During subsequent therapies
**Patients with CNS metastases, *n***	**25**	**47**	**24**	**4**
**Characterization of CNS metastases, *n* (%)**	**24 (100)**	**47 (100)**	**23 (100)**	**4 (100)**
Measurable/Non-measurable	16 (66.7)/5 (20.8)	21 (44.7)/17 (36.2)	10 (43.5)/9 (39.1)	1 (25)/1 (25)
Symptomatic/Non-symptomatic	12 (50)/9 (37.5)	23 (48.9)/21 (44.7)	15 (65.2)/6 (26.1)	3 (75)/0 (0)
Solitary/Multiple	11 (45.8)/10 (41.7)	9 (19.1)/27 (57.4)	3 (13)/15 (65.2)	1 (25)/2 (50)
Presence/absence of LM carcinomatosis	1 (4.2)/13 (54.2)	4 (8.5)/23 (48.9)	2 (8.7)/13 (56.5)	0 (0)/0 (0)
**Patients treated for CNS metastases**	**13 (52)**	**14 (29.8)**	**12 (50)**	**2 (50)**
**Patients treated locally, *n* (%)**	**12 (92.3)**	**13 (93)**	**6 (50)**	**2 (100)**
Whole-brain radiotherapy	6 (50)	8 (61.5)	5 (83.3)	2 (100)
Radiosurgery	6 (50)	5 (38.5)	1 (16.7)	0 (0)
Brain surgery plus radiotherapy	0 (0)	0 (0)	0 (0)	0 (0)
Brain surgery	2 (16.7)	0 (0)	0 (0)	0 (0)
**Duration of radiotherapy (days)**				
Mean (SD; 95% CI)	19 (15.3; 05–37.9)	9.5 (4.5; 5.7–13.3)	11 (4.3; 4.1–17.9)	11 (2.8; –14.4–36.4)
Median (IQR; min, max)	11 (10–30; 4, 40)	8.5 (6–13.5; 4, 16)	12 (8–14; 5, 15)	11 (9–13; 9, 13)
**Patients treated with corticosteroids, *n* (%)**	**3 (23.2)**	**5 (35.7)**	**10 (83.3)**	**1 (50)**
**Corticosteroid dose (mg)**				
Mean (SD; 95% CI)	3.5 (3.5; –28.3–35.3)	4.7 (3; –0–9.5)	8.1 (8.9; 1.2–15)	1 (--; -- - --)
Median (IQR; min, max)	3.5 (1–6; 1, 6)	5 (2.5–7; 1, 8)	6 (2–8; 1, 30)	1 (1–1; 1, 1)
**DOT with corticosteroids (months)**				
Mean (SD; 95% CI)	13.2 (18.5; –153.2–179.7)	2.2 (2.3; –0.7–5)	5.9 (7.4; 0.2–11.7)	0.2 (--; -- - --)
Median (IQR; min, max)	13.2 (0.1–26.3; 0.1, 26.3)	1.4 (0.8–1.7; 0.8, 6.2)	2.6 (0.5–9.5; 03, 19.8)	0.2 (0.2–0.2; 0.2, 0.2)

Abbreviations: CI: confidence interval; CNS: central nervous system; DOT: duration of treatment; IQR: interquartile range (25–75); LM: leptomeningeal; SD: standard deviation.

Supplementary Table 3: First and best response achieved after management of CNS progression during alectinib treatment

	Total	Radiotherapy	Non-radiotherapy	P[2a]
**First response achieved, *n* (%)**	**21 (100)**	**4 (100)**	**17 (100)**	0.14
Complete response (CR)	2 (9.5)	1 (25)	1 (5.9)	
Partial response (PR)	4 (19)	2 (50)	2 (11.8)	
Stable disease (SD)	8 (38.1)	0 (0)	8 (47.1)	
Progressive disease (PD)	6 (28.6)	1 (25)	5 (29.4)	
Not evaluable (NE)	1 (4.8)	0 (0)	1 (5.9)	
**Time to first response (months)**	**Valid *N* (20)**	**Valid *N* (4)**	**Valid *N* (16)**	
Mean (SD; 95% CI)	4.1 (4.6; 1.9–6.2)	6.8 (6.3; –3.2–16.8)	3.4 (4; 1.2–5.5)	0.48
Median (IQR; min, max)	2.6 (1.3–3.5; 0.2, 17.1)	5.8 (1.7–11.9; 1.1, 14.5)	2.6 (1.3–3.4; 0.2, 17.1)	
**Best response achieved, *n* (%)**	**21 (100)**	**4 (100)**	**17 (100)**	
Complete response (CR)	1 (4.8)	0 (0)	1 (5.9)	0.21
Partial response (PR)	5 (23.8)	3 (75)	2 (11.8)	
Stable disease (SD)	9 (42.9)	1 (25)	8 (47.1)	
Progressive disease (PD)	5 (23.8)	0 (0)	5 (29.4)	
Not evaluable (NE)	1 (4.8)	0 (0)	1 (5.9)	
**Overall response rate (ORR), *n* (%)**	**21 (100)**	**4 (100)**	**17 (100)**	
CR + PR	6 (28.6)	3 (75)	3 (17.6)	0.05
SD + PD + NE	15 (71.4)	1 (25)	14 (82.4)	
**Disease control rate (DCR), *n* (%)**	**21 (100)**	**4 (100)**	**17 (100)**	
CR + PR	15 (71.4)	4 (100)	11 (64.7)	0.28
SD + PD + NE	6 (28.6)	0 (0)	6 (35.3)	
**Time to best response (months)**	**Valid *N* (20)**	**Valid *N* (4)**	**Valid *N* (16)**	
Mean (SD; 95% CI)	4.5 (5.3; 2–7)	8.7 (8.4; –4.7–22.1)	3.4 (4; 1.3–5.6)	0.48
Median (IQR; min, max)	2.6 (1.5–3.5; 0.2, 18.6)	7.5 (1.7–15.6; 1.1, 18.6)	2.6 (1.5–3.4; 0.2, 17.1)	
**Duration of response (months)**	**Valid *N* (6)**	**Valid *N* (3)**	**Valid *N* (3)**	
Mean (SD; 95% CI)	11 (7.5; 3.1–18.9)	5.2 (1.8; 0.8–9.6)	16.8 (6.1; 1.5–32.1)	0.03
Median (IQR; min, max)	10.2 (4.4–13.4; 4, 24)	4.4 (4–7.2; 4, 7.2)	13.4 (13.1–23.9; 13.1, 24)	

[a]Fisher's exact test for categorical variables; Two-sample *t*-tests or Mann–Whitney *U* test for continuous variables. Non-radiotherapy was considered if the CNS treatment was "Radiosurgery" or "Brain surgery" or "Other", as well as patients with CNS metastases during alectinib treatment but without CNS treatment reported during this period. Abbreviations: CI: confidence interval; CNS: central nervous system; IQR: interquartile range (25–75); SD: standard deviation.

Supplementary Table 4: Type of adverse events (AE) according to MedDRA reported during treatment with alectinib in the safety population

**Total no. of patients with AE, *n* (%)**	**43 (35.8)**
**Blood and lymphatic system disorders**	**1 (0.8)**
Thrombocytopenia	1 (0.8)
**Gastrointestinal disorders**	**1 (0.8)**
Diarrhea	1 (0.8)
Dysphagia	1 (0.8)
Pancreatitis	1 (0.8)
Vomiting	2 (1.7)
**General disorders and administration site conditions**	**11 (9.2)**
Asthenia	3 (2.5)
Fatigue	3 (2.5)
General physical health deterioration	1 (0.8)
Inflammation	1 (0.8)
Edema	1 (0.8)
Pyrexia	1 (0.8)
Sudden death	1 (0.8)
Xerosis	1 (0.8)
**Hepatobiliary disorders**	**3 (2.5)**
Hepatotoxicity	2 (1.7)
Hypertransaminasemia	1 (0.8)
**Infections and infestations**	**2 (1.7)**
Respiratory tract infection	1 (0.8)
Septic shock	1 (0.8)
**Investigations**	**13 (10.8)**
Alanine aminotransferase increased	1 (0.8%)
Aspartate aminotransferase increased	1 (0.8%)
Blood bilirubin increased	3 (2.5%)
Blood creatine phosphokinase increased	1 (0.8%)
Gamma-glutamyltransferase increased	2 (1.7%)
General physical condition abnormal	2 (1.7%)
Heart rate decreased	1 (0.8%)
Transaminases increased	3 (2.5%)
**Metabolism and nutrition disorders**	**2 (1.7)**
Enzyme abnormality	1 (0.8)
Obesity	1 (0.8)
**Musculoskeletal and connective tissue disorders**	**6 (5)**
Arthralgia	1 (0.8%)
Muscle spasms	1 (0.8%)
Musculoskeletal chest pain	1 (0.8%)
Myalgia	1 (0.8%)
Myositis	2 (1.7%)
Neck pain	1 (0.8%)

**Nervous system disorders**	**5 (4.2)**
Cerebrovascular accident	1 (0.8%)
Dizziness	1 (0.8%)
Dysesthesia	1 (0.8%)
Nervous system disorder	1 (0.8%)
Paresthesia	1 (0.8%)
**Respiratory, thoracic and mediastinal disorders**	**9 (7.5)**
Asthma	1 (0.8)
Catarrh	1 (0.8%)
Cough	2 (1.7%)
Dyspnea	5 (4.2%)
Pneumonitis	1 (0.8%)
Pulmonary embolism	1 (0.8%)
Respiratory failure	1 (0.8%)
**Skin and subcutaneous tissue disorders**	**1 (0.8)**
Palmar-plantar erythrodysesthesia syndrome	1 (0.8)
**Vascular disorders**	**1 (0.8)**
Superior vena cava syndrome	1 (0.8)

[a]Terms coded using MedDRA Dictionary, version 23.0 (SOC and PT name).
